# Identification of *FBXL4* as a Metastasis Associated Gene in Prostate Cancer

**DOI:** 10.1038/s41598-017-05209-z

**Published:** 2017-07-11

**Authors:** Elzbieta Stankiewicz, Xueying Mao, D. Chas Mangham, Lei Xu, Marc Yeste-Velasco, Gabrielle Fisher, Bernard North, Tracy Chaplin, Bryan Young, Yuqin Wang, Jasmin Kaur Bansal, Sakunthala Kudahetti, Lucy Spencer, Christopher S. Foster, Henrik Møller, Peter Scardino, R. Tim Oliver, Jonathan Shamash, Jack Cuzick, Colin S. Cooper, Daniel M. Berney, Yong-Jie Lu

**Affiliations:** 1 Molecular Oncology, Barts Cancer Institute, Queen Mary University of London, Charterhouse Square, London EC1M 6BQ UK; 2 The Robert Jones and Agnes Hunt Orthopaedic Hospital, Department of Pathology, Oswestry, Shropshire SY10 7AG UK; 3 Cancer Research UK Centre for Epidemiology, Mathematics and Statistics, Wolfson Institute of Preventive Medicine, Queen Mary University of London, London, EC1 6BQ UK; 4 Centre for Haemato-Oncology, Barts Cancer Institute, Queen Mary University of London, Charterhouse Square, London EC1M 6BQ UK; 5 Division of Cellular and Molecular Pathology, University of Liverpool, Liverpool, L69 3BX UK; 6 King’s College London, Cancer Epidemiology and Population Health, London, SE1 9RT UK; 7 Department of Urology, Memorial Sloan Kettering Cancer Center, New York, NY 10065 USA; 8 School of Medicine, University of East Anglia, Norwich, NR4 7TJ UK; 9 HCA Pathology Laboratories, Shropshire House, Capper Street, London, WC1E6JA UK

## Abstract

Prostate cancer is the most common cancer among western men, with a significant mortality and morbidity reported for advanced metastatic disease. Current understanding of metastatic disease is limited due to difficulty of sampling as prostate cancer mainly metastasizes to bone. By analysing prostate cancer bone metastases using high density microarrays, we found a common genomic copy number loss at 6q16.1–16.2, containing the *FBXL4* gene, which was confirmed in larger series of bone metastases by fluorescence *in situ* hybridisation (FISH). Loss of *FBXL4* was also detected in primary tumours and it was highly associated with prognostic factors including high Gleason score, clinical stage, prostate-specific antigen (PSA) and extent of disease, as well as poor patient survival, suggesting that *FBXL4* loss contributes to prostate cancer progression. We also demonstrated that *FBXL4* deletion is detectable in circulating tumour cells (CTCs), making it a potential prognostic biomarker by ‘liquid biopsy’. *In vitro* analysis showed that *FBXL4* plays a role in regulating the migration and invasion of prostate cancer cells. FBXL4 potentially controls cancer metastasis through regulation of ERLEC1 levels. Therefore, *FBXL4* could be a potential novel prostate cancer suppressor gene, which may prevent cancer progression and metastasis through controlling cell invasion.

## Introduction

Prostate cancer is the most common cancer and second leading cause of cancer death among Western men^1^. Many prostate cancers are indolent, meaning patients die with, rather than of cancer. It is challenging to differentiate between indolent and life-threatening disease. Many indolent cancers are treated unnecessarily with a significant reduction in quality of life. However, if untreated, prostate cancer may metastasize, and become incurable^2^. Understanding of the genetic drivers of cancer progression to metastatic disease is critical to improve the survival rate of prostate cancer patients.

Unlike other cancers, prostate cancer creates osteoblastic rather than osteolytic bone deposits^3, 4^, which makes it particularly relevant to investigate its mechanisms of bone metastasis. However, little is known of the genetic changes associated with prostate cancer bone metastasis, partly due to the difficulty in obtaining samples from the bone. Many genome-wide studies examined genetic alterations only in localised disease^5–8^. The majority of microarray and genome-wide sequencing studies of advanced prostate cancer only analysed tissue from primary tumours or local lymph nodes rather than from distant metastatic sites^9–11^. Only a few studies have examined genetic changes in advanced lethal prostate cancer at metastatic sites, which included only a small proportion of metastases from the bone^12–17^.

To identify genomic alterations, in particular genes associated with prostate cancer bone metastasis, we performed microarray analysis of fresh frozen prostate cancer bone metastasis samples and found 6q16.1–16.2, containing *FBXL4* gene, as commonly deleted genomic region of bone metastatic prostate cancer. Further study revealed that *FBXL4* was also deleted in a proportion of early stage prostate cancer cases, which was associated with poor prognosis and reduced survival. We also detected *FBXL4* in circulating tumour cells (CTCs). Functional analysis of *FBXL4* in prostate cancer cell lines suggested its involvement in cell migration and invasion, potentially through regulation of the levels of Endoplasmic Reticulum Lectin 1 (ERLEC1) protein, a regulator of cellular stress-response and promoter of metastatic cell survival^18^.

## Results

### Loss of 6q16 and down-regulation of *FBXL4* is commonly found in prostate cancer bone metastases

**Figure 1:**
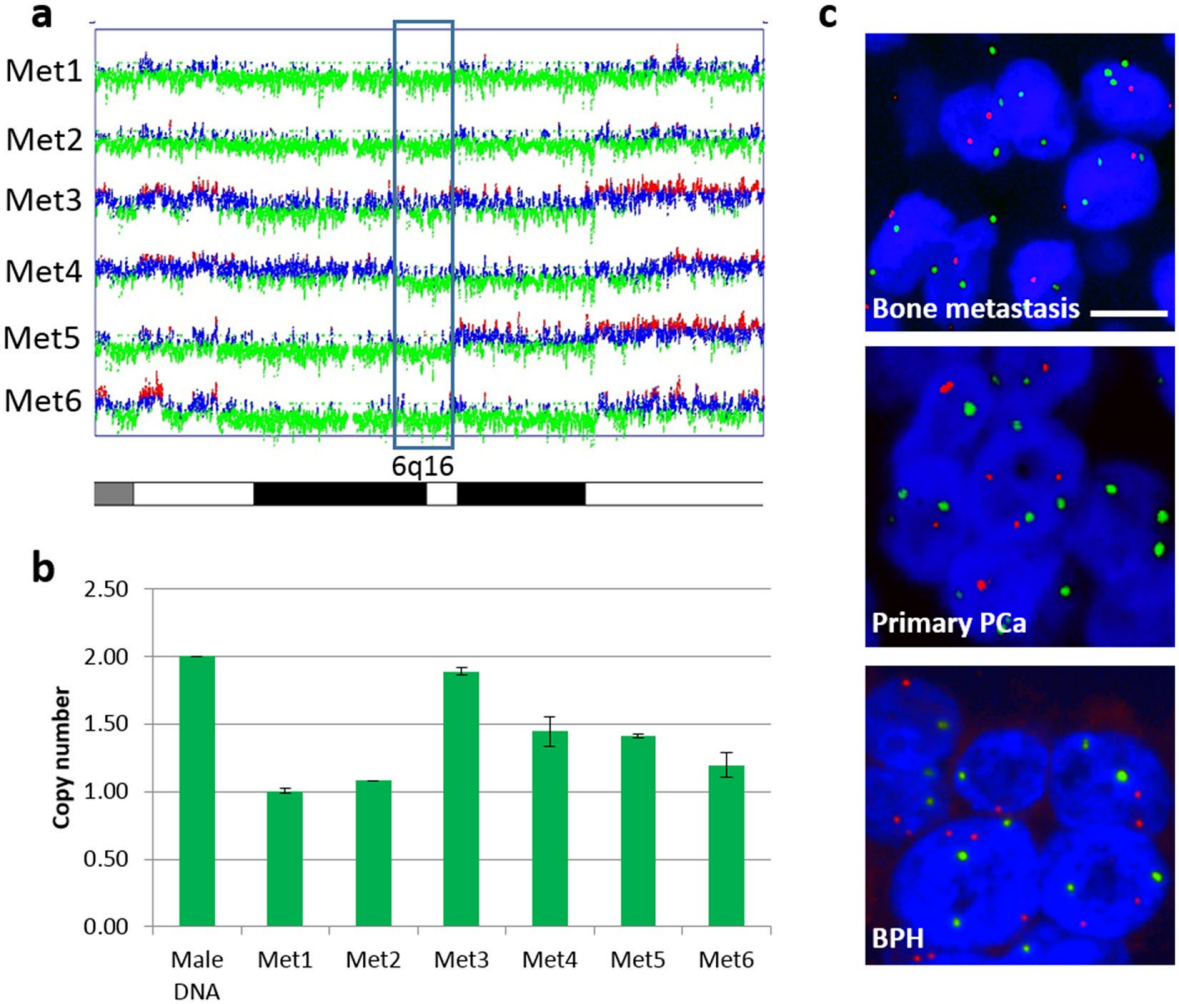
Common loss of 6q16 including *FBXL4* locus is present in prostate cancer samples. (**a**) Minimum overlapping region of copy number loss at 6q16.1–16.2 (blue box) in six fresh frozen prostate cancer bone metastases detected by Affymetrix SNP array 6.0. Heterozygous deletion is present in all six samples. SNPs with normal copy number are depicted in blue, loss in green and gain in red. (**b**) SNP array results were confirmed with copy number analysis of 6q16.1–16.2 at *FBXL4* locus by TaqMan DNA copy number assay. Heterozygous *FBXL4* loss was confirmed in 5/6 samples. Sample 3 (Met 3) with least obvious deletion on SNP array shows close to normal *FBXL4* copy number by qPCR, indicating a small proportion of cells with this deletion. Error bars: SD; n = 3. (**c**) Representative FISH images of *FBXL4* region in prostate clinical samples. *FBXL4* copy number loss in a metastatic and primary FFPE prostate cancer sample is visible in most nuclei (blue) as a reduction in number of red signals (FBXL4) in relation to green (control) signals. Neutral copy number in a BPH case is reflected by equal number of red (FBXL4) and green (control) signals per nucleus. Scale bar: 10 µm.

We initially analysed the genomic alterations in six fresh frozen samples of prostate cancer bone metastases by Affymetrix single nucleotide polymorphism (SNP) array 6.0 and found chromosomal copy number loss commonly affecting 6q14.1–22.32 with minimum overlapping region at 6q16.1–16.2 in 5/6 cases (Fig. 1a), suggesting that this genetic region harbours a potential tumour suppressor gene (TSG). 6q16.1–16.2 contains only two genes: a widely expressed *FBXL4*, a member of the F-box protein family, and *BRN2*, a master regulator of neuronal differentiation and a member of the mammalian class III POU transcription factor family, mainly expressed in the developing central nervous system^19^. 6q16.1–16.2 copy number loss at *FBXL4* gene location was confirmed in those samples by TaqMan DNA copy number analysis (Fig. 1b). Additional analysis of *FBXL4* genomic region in a bigger cohort of formalin-fixed, paraffin-embedded (FFPE) prostate cancer bone metastases samples by fluorescence *in situ* hybridisation (FISH) detected loss of this chromosomal region in 11/23 (47.8%) cases (Fig. 1c), indicating that *FBXL4* gene may be a gene commonly down-regulated in prostate cancer bone metastasis. Heterozygous loss of 6q16.1–16.2 also existed in both metastatic PC3 and DU145 prostate cancer cell lines, but not in less aggressive 22RV1, LNCaP and VCaP cell lines. We analysed *FBXL4* RNA expression levels by quantitative reverse transcription polymerase chain reaction (q-RT-PCR) in five bone metastases and four primary prostate cancer samples and found a significant reduction in *FBXL4* expression in bone metastases and primary cancer samples compared to six benign prostatic hyperplasia (BPH) samples (p = 0.001, Kruskal-Wallis test, Supplementary Figure 1a). The reduced levels of *FBXL4* expression were also present in both cell lines with 6q16.1–16.2 loss (PC3 and DU145) when compared to cell lines without the loss (22RV1, LNCaP and VCaP cells, Supplementary Figure 1b). On the contrary, expression of *BRN2*, the other gene located within 6q16 deletion, did not correlate with cancer progression (p = 0.226, Kruskal-Wallis test) when tested in the same clinical samples (Supplementary Figure 1c). All these findings suggest that *FBXL4* could be the putative TSG located at the deleted region, whose loss may result in prostate cancer progression.

### Loss of *FBXL4* genomic region is present in primary tumours and correlates with advanced prostate cancer disease and prostate cancer specific death

To investigate if loss of *FBXL4* genomic region also occurs at early stage of prostate cancer development, we analysed 145 primary cancer samples by FISH and detected *FBXL4* genomic loss in 20 cases (13.8%), but not in any of the 55 BPH cases used as non-neoplastic controls (Fig. 1c). The relatively low frequency of *FBXL4* genomic region loss in primary tumours and its significant increase in frequency in metastatic samples (p = 0.0003, Supplementary Table 1) further supports the hypothesis that *FBXL4* is a gene that suppresses prostate cancer metastatic progression.

**Figure 2:**
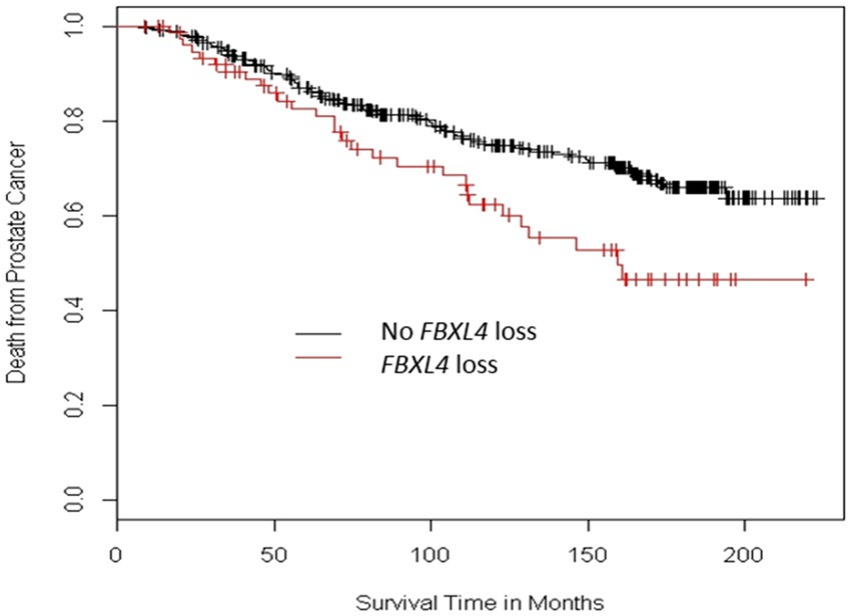
*FBXL4* loss is significantly associated with decreased prostate cancer specific survival in univariate analysis. Kaplan–Meier survival curves represent conservatively managed localised prostate cancers with or without *FBXL4* loss. Presence of *FBXL4* loss is univariately linked to higher rate of prostate cancer specific death (Cox model, HR = 1.738 (1.147–2.633), p = 0.009). Cohort size: 447 cases, with 77 of *FBXL4* loss positive.

To further elucidate the role of *FBXL4* in the natural history of prostate cancer, we investigated *FBXL4* copy number status in the TransAtlantic Prostate Group (TAPG) cohort of conservatively managed localised prostate adenocarcinomas diagnosed by a transurethral resection of the prostate (TURP) to assess *FBXL4* loss as a potential cancer prognostic biomarker. *FBXL4* loss was present in 77/447 (17%) cases successfully analysed by FISH, which is consistent with the above data from a separate cohort of primary prostate cancers. Interestingly, *FBXL4* loss strongly correlated with high Gleason score and clinical stage (p = 0.0000025 and 0.0005, respectively, Table 1) as well as with prostate-specific antigen (PSA) and extent of the disease (p = 0.0002 and 0.00005, respectively). More importantly, loss of *FBXL4* was significantly associated with death from prostate cancer (HR 1.7, p = 0.009, Fig. 2) by univariate analysis. However, since loss of *FBXL4* was strongly correlated with other poor prognostic factors such as PSA, as expected, the correlation with patient outcome was not significant in multivariate analysis when Gleason score, baseline PSA and extent of the disease were included in the analysis model (Supplementary Table 2). Therefore, loss of *FBXL4* in prostate cancer tissue, while significantly correlating with advanced disease, was not an independent prognostic factor for patient outcome.

**Table 1:** Correlation of *FBXL4* loss with Gleason scores and clinical stages.

Parameter	No *FBXL4* loss	*FBXL4* loss	p value (*X* ^2^ test)
Gleason score < 7	197	20	0.000003
Gleason score = 7	97	22	
Gleason score > 7	76	35	
Clinical stage T1	104	10	0.0005
Clinical stage T2	83	32	
Clinical stage T3	40	16	
Clinical stage Tx	63	11

**Figure 3:**
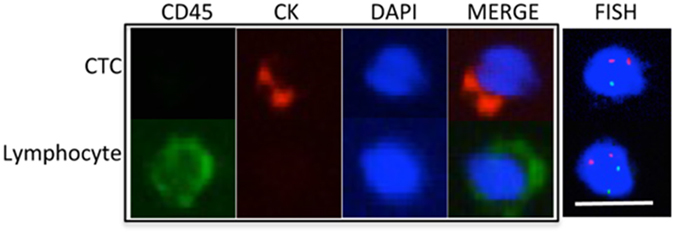
Representative FISH image shows the deletion of *FBXL4* in a CTC. CTC and lymphocyte were determined by immunofluorescence staining (left four panels). By FISH, two chromosome 1 paracentromere (red) regions were detected, but only one *FBXL4* (green) was found in the CTC (heterozygous loss), while there were two copies of both chromosome 1 paracentromere and *FBXL4* regions in the lymphocyte (no copy number change). Scale bar: 10 µm.

As blood samples are more accessible than tissue biopsy for monitoring tumour progression^20^, we investigated whether *FBXL4* loss is detectable in prostate cancer circulating tumour cells (CTCs). In six of seven blood samples from patients with bone metastatic prostate cancer (Gleason score 4 + 4 or 5 + 5), we detected *FBXL4* deletion in CTCs (Fig. 3 and Supplementary Table 3). *FBXL4* loss was also found in CTCs from a patient with lymph node metastasis only, and in a non-metastatic T3 prostate cancer case with high Gleason score 4 + 4. The remaining two patients tested had lower Gleason score (3 + 3 and 4 + 3) and no CTCs with *FBXL4* loss (Supplementary Table 3).

### Manipulation of *FBXL4* expression level affects cell migration and invasion without affecting cell viability

**Figure 4:**
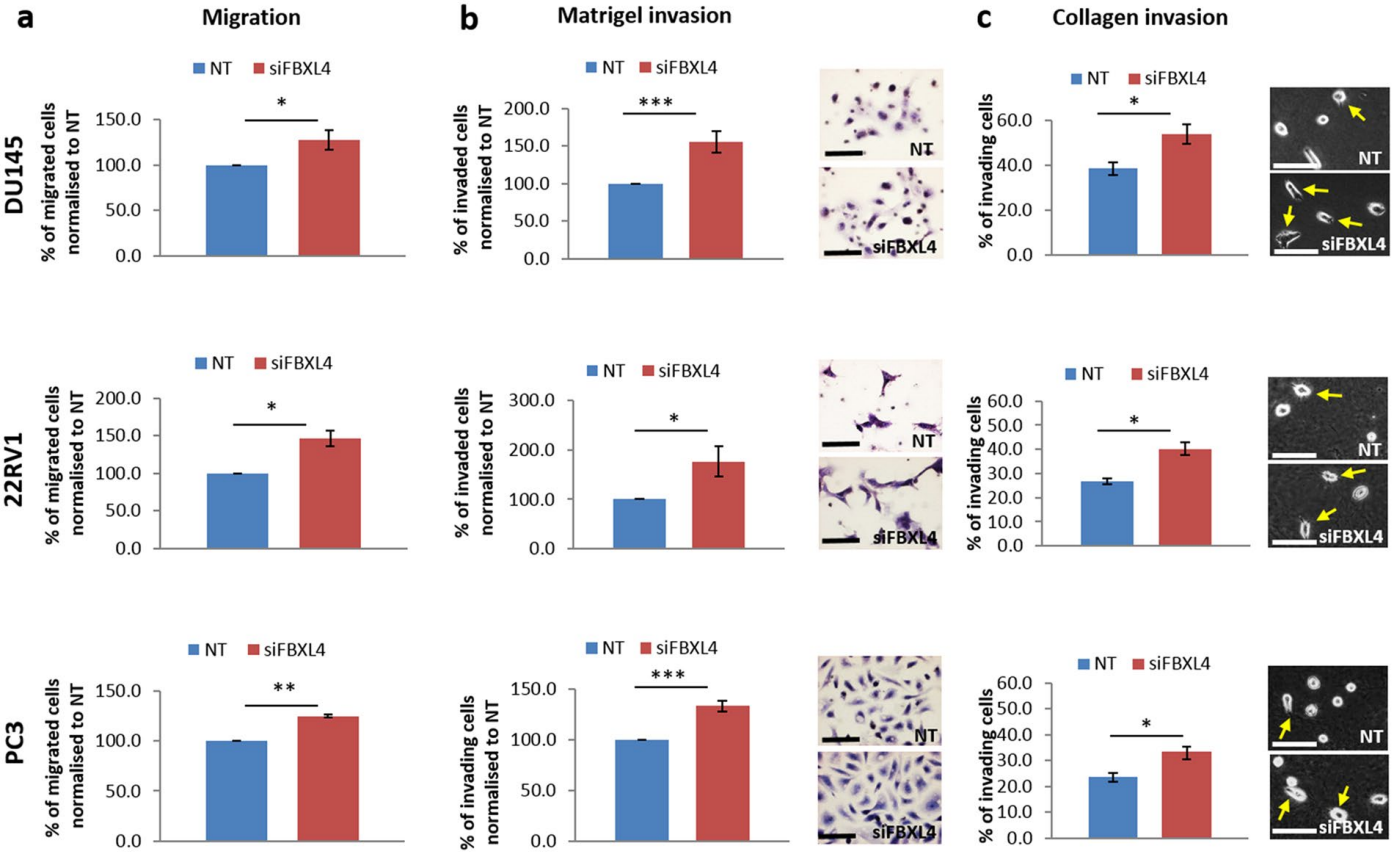
*FBXL4* knockdown leads to increased prostate cancer cell migration and invasion in DU145, 22RV1 and PC3 cells. (**a**) Prostate cancer cells transwell migration assay showing increased cell migration with *FBXL4* knockdown. (**b**) Prostate cancer cell invasion through Matrigel. Exemplary pictures on the right show increased number of cells invaded through the Matrigel after *FBXL4* knockdown. Invaded cells were formalin-fixed and stained with haematoxylin for easy visualisation and counting. (**c**) Prostate cancer cell migration/invasion on collagen. Cells were seeded onto collagen (2 mg/ml) as a single cell suspension and cells with pseudopodia-like extensions (arrows) were counted as migrating/invading cells^52^. In all experiments, cells were allowed migration/invasion for 24 h. Number of migrating/invading cells was determined by counting the cells in at least 10 random fields in each of three independent biological experiments. NT, cells transfected with non-targeting siRNA; siFBXL4, cells transfected with *FBXL4* siRNA; arrows, migrating/invading cells. Error bars show the SDs in n = 3 expereiments. p-values were calculated relative to cells transfected with non-targeting siRNA. *p < 0.05, **p < 0.01 and ***p < 0.001 (two-tailed Student’s t-test). Scale bar: 50 µm.

To functionally investigate how *FBXL4* contributes to prostate cancer progression, we knocked it down with small interfering RNAs (siRNA) in DU145, 22RV1 and PC3 cells (Supplementary Figure 2a). Knockdown of *FBXL4* had no effect on cell viability (Supplementary Figure 2b) but led to a significant increase in cell migration (p = 0.04, 0.02 and 0.002, respectively) for all the three cell lines (Fig. 4a and Supplementary Table 4).

We also investigated if *FBXL4* affects cell invasion ability. *FBXL4* knockdown increased Matrigel invasion in all prostate cancer cell lines (p = 0.001, 0.04 and 0.0001 for DU145, 22RV1 and PC3, respectively; Fig. 4b). As loss of *FBXL4* was found in prostate cancer bone metastases and about 95% of bone organic matter consists of collagen, we further analysed cell motility/invasion in the context of a bone microenvironment by seeding prostate cancer cells as a single cell suspension on top of collagen gels. As invading cells produce cellular protrusions (pseudopodia) we counted the cells with pseudopodia-like extensions and detected significant motility/invasion increase with *FBXL4* knockdown compared to cells transfected with non-targeting siRNA in all three cell lines tested (p = 0.01, 0.01 and 0.03 for DU145, 22RV1 and PC3 cells respectively, Fig. 4c and Supplementary Table 4). These results suggest that *FBXL4* knockdown also leads to a modest but consistent increase in cell invasion into collagen.

**Figure 5:**
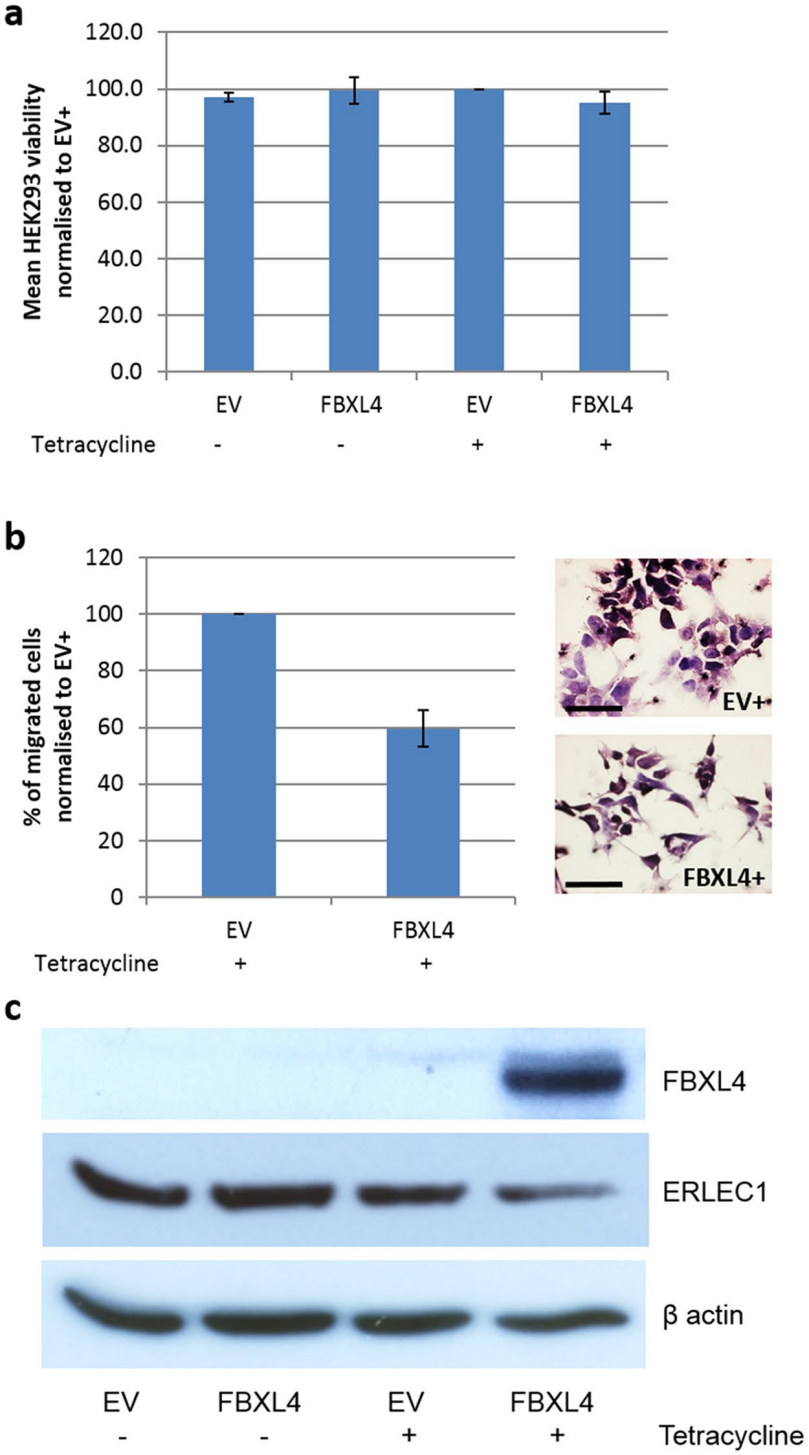
*FBXL4* overexpression did not affect cell viability but resulted in reduced HEK293 cell migration and ERLEC1 downregulation. (**a**) 72 h induction of *FBXL4* overexpression with 1 µg/ml tetracycline does not affect HEK293 cell viability when compared to control HEK293 cells containing empty vector. (**b**) HEK293 cells overexpressing *FBXL4* show reduced migration when compared with control HEK293 cells containing empty vector. Graph on the left represents average results from three independent experiments. Exemplary pictures on the right show reduced number of cells migrated through the transwell membrane after *FBXL4* overexpression. Migrated cells were formalin-fixed and stained with haematoxylin for easy visualisation and counting. Scale bar: 50 µm. (**c**) Western blots showing that FBXL4 overexpression leads to downregulation of ERLEC1 protein levels in HEK293 cells. EV, control cells with empty expression vector; FBXL4, HEK293 cells transfected with expression vector containing *FBXL4*. ‘−‘, no tetracycline treatment; ‘+’, 1 µg/ml tetracycline treatment for 72 h. Error bars: SD; n = 3.

To confirm that *FBXL4* expression levels influence cell migration, we used a gene overexpression approach to generate HEK293 cells with tetracycline-inducible *FBXL4* expression. Gene overexpression was confirmed at both RNA and protein levels (Supplementary Figure 3a and b). Overexpression of *FBXL4* had no effect on cell viability (Fig. 5a), but as expected led to significant (40.4%) reduction in cell migration when compared to control cells transfected with an empty vector (p = 0.008, Fig. 5b). As the acquisition of a motile and invading phenotype is often linked to the epithelial–mesenchymal transition (EMT) and many F-box proteins are involved in regulation of this process^21^, we investigated the impact of FBXL4 overexpression on the levels of the following proteins linked to EMT and cell movement, including mesenchymal markers – vimentin and N-cadherin, epithelial marker CK18, and focal adhesion kinase (FAK). Western Blotting (WB) analysis showed no difference in their expression in HEK293 cells overexpressing FBXL4 when compared to control cells (Supplementary Figure 4) indicating that FBXL4 may have limited effect on the expression of these proteins.

### ERLEC1 is a FBXL4 degradation target

As ERLEC1 protein was previously reported as a binding partner of FBXL4^22^, we investigated the relationship between these two proteins using *in vitro* FBXL4 overexpression model. Anti-ERLEC1 antibody specificity was confirmed by western blot in HEK293 cells with ERLEC1 downregulation (Supplementary Figure 5). Overexpression of FBXL4 in HEK293 cells led to downregulation of ERLEC1 protein when compared to control cells without FBXL4 overexpression (Fig. 5c). Overexpression of FBXL4 in HEK293 cells led to the reduction in ERLEC1 protein level, suggesting that ERLEC1 is targeted by FBXL4 for degradation.

## Discussion

Bone metastasis is significantly associated with death from prostate cancer and also leads to major and debilitating symptoms including ‘bone pain’^3, 4^. Up to 90% of patients dying from prostate cancer have skeletal metastases^23^. Identifying genetic changes contributing to cancer metastasis to the bone is critical to reduce both prostate cancer mortality and morbidity. However, most of the prostate cancer genomic studies have been performed on prostatectomy specimens, including studies of metastatic cancers^9–11^. Due to the difficulty in obtaining tissue from bone, very few cases of metastases from the bone have been included in previous studies^12, 13, 15^. Identification of driver genes for bone metastasis will help the development of biomarkers and therapeutic targets for the palliation and possible cure of disease at this site.

By genomic profiling of prostate cancer bone metastatic samples, we found a common genomic deletion at 6q16.1–16.2 and through further investigations we identified the *FBXL4* gene, located within this 6q16.1–16.2 region, as the putative TSG in regulating prostate cancer bone metastases. The only other gene located within the deleted 6q16.1–16.2, *BRN2*, is a leading regulator of neuronal differentiation, mainly expressed during development in cells originated from neural crest. *BRN2* is known as an oncogene in melanomas (originating from melanocytes derived from neural crest cells)^24^, neuroendocrine small cell lung cancers (SCLCs)^25^, where its overexpression correlates with cell invasiveness and metastatic capacity^24^, and lethal neuroendocrine prostate cancers^26^. In our clinical samples, only *FBXL4* but not *BRN2* expression correlated with prostate cancer progression. Thus it is unlikely for *BRN2* to be a candidate driver gene for this deletion. We also demonstrated that *FBXL4* was not only commonly deleted in bone metastasis samples but also in early stage cancers, although at significantly lower frequency than in the metastatic cancers. The detection of *FBXL4* deletion in localised prostate cancer is consistent with previous studies, reporting 6q16 deletion as one of the frequent genomic changes^27, 28^. These findings further support loss of *FBXL4* as a driver genetic event in prostate cancer progression to bone metastasis and also make the alteration of this gene a potential biomarker to predict prostate cancer progression and outcome.

Consistent with the association of *FBXL4* loss with prostate cancer metastasis, we demonstrated that *FBXL4* regulates cell motility and invasiveness by both, *FBXL4* knockdown and overexpression. Although the functional effect of *FBXL4* on migration and invasion of prostate cancer cell lines in our study is low, *FBXL4* downregulation consistently led to the increase while overexpression resulted in the decrease of cell migration and invasiveness. It is not unusual that genes with apparent *in vivo* tumour suppressor or oncogenic capacity have relatively small or moderate effect in *in vitro* assays including a F-box family protein, FBXW7, for its role in cell migration and invasion^29–31^. It is also known that the effect depends on assay and cell line used^32^. Therefore, the consistency of our results obtained in different cell lines with varied invasiveness, in addition to our clinical sample data, supports that *FBXL4* regulates prostate cell migration and invasiveness.

FBXL4 is a member of the F-box protein family, which is part of E3 ubiquitin protein ligase complex called the SKP1-CUL1-F-box (SCF) complex, involved in protein ubiquitination and degradation^33^. The exact role of *FBXL4* gene is presently unknown. Apart from the link between rare autosomal recessive *FBXL4* loss-of-function and splice mutations and early-onset mitochondrial encephalomyopathy^34–36^, there are no reports on *FBXL4* function and potential involvement in cancer. However through substrate downregulation, F‑box proteins regulate diverse biological pathways that control cell growth, division, signalling responses, EMT transition, survival and death^37^. Unsurprisingly, more and more evidence emerges about deregulation of these proteins in human cancer and their impact on cancer development, progression and metastasis^38, 39^. F-box protein family members are known to act as oncogenes (e.g. *SKP2*) or TSG (e.g. *FBXW7*) often through their ability to control degradation of cell cycle proteins and cell proliferation^40^ or through induction of EMT^29, 41^. However, changing *FBXL4* expression level did not affect cell proliferation or protein levels of EMT markers that we examined, suggesting that *FBXL4* may control proteins in different cellular pathways, which contribute to prostate cancer invasion and metastasis. Involvement of F-box proteins in controlling cancer spread and metastasis is common and can encompass different mechanisms such as resistance to anoikis, a type of detachment-induced apoptosis affecting tumour cells detached from primary mass (*SKP2* oncogene ref. 42), inhibition of niche formation at distant metastatic site (*FBXW7* TSG ref. 43) or modulates cellular stress response and metastatic potential (*FBXW7* TSG ref. 44). Further mechanistic investigations are required.

Recent study through proteomics based approach, aiming at the identification of candidate substrates and stably interacting proteins for a range of F-box family members, discovered several proteins potentially interacting with FBXL4^22^. One of them was a luminal resident protein of the endoplasmic reticulum, ERLEC1 and its direct interaction with FBXL4 was confirmed by immunoprecipitation/western blot. ERLEC1 is a molecular chaperone that plays a role in endoplasmic reticulum stress response^45^. It is frequently overexpressed in human cancers and was identified in lung cancer as a novel cancer invasion and metastasis-related gene controlling the response to hypoxia and ER stress^18^. Ectopic expression of ERLEC1 in lung cancer cells increased their tolerance to hypoxia and endoplasmic reticulum stress, protecting the cells from apoptosis, while ERLEC1 knockdown resulted in reduction in metastasis in a mouse xenograft model^18^. In agreement with these findings, we demonstrated that overexpression of FBXL4 led to the downregulation of ERLEC1. Thus *FBXL4* may prevent cancer metastasis through regulation of ERLEC1 protein levels.

The biological role of *FBXL4* in suppressing cancer progression to advanced disease was also confirmed by the strong associations between *FBXL4* loss and high Gleason score, advanced clinical stage, high PSA and large disease volume in a cohort of conservatively managed localised prostate cancer. The loss of *FBXL4* was significantly associated with poor patient survival (HR 1.7, p = 0.009) although Gleason score, clinical T stage and serum PSA levels were stronger prognostic indicators. Gleason score is a local invasiveness indicator for prostate cancer. The strong correlation of *FBXL4* loss and Gleason score is consistent with our *in vitro* observation that *FBXL4* is associated with cancer cell migration and invasion. Loss of *FBXL4* may contribute to cancer cell distribution patterns associated with higher Gleason scores and therefore are both associated with bone metastasis and poor prognosis.

We demonstrated that loss of *FBXL4* can be detected in prostate cancer CTCs, making it possible to analyse *FBXL4* status in CTCs to monitor the risk of bone metastasis. CTCs from peripheral blood provide a ‘liquid biopsy’, making it possible to frequently and in real time monitor disease progression and response to therapies.

Although mutations in *FBXL4* may occur, they are uncommon (https://icgc.org/ and http://cancer.sanger.ac.uk/) and in a recent exome sequencing study of 150 metastatic castration-resistant prostate cancer, only two point mutations were found in *FBXL4*, while deletion of this genomic region was detected in 20% of samples^16^. Loss of *FBXL4* copy number may be the main mechanism that leads to low expression of this gene and resulting haplo-insufficiency may contribute to cancer progression. In addition to bone metastasis, 6q16 deletion has been commonly observed in prostate cancer metastases to other sites in the body^14, 16, 27, 28^, making it a more attractive biomarker and therapeutic target. Moreover, 6q16 deletion has also been frequently found in other human cancers, such as breast, lung, colon, ovarian cancers and malignant melanoma^46^, where loss of *FBXL4* may be a driver for cancer progression. Using *FBXL4* copy number change as a prognostic marker and targeting the downstream pathways of FBXL4 for cancer treatment may have a much broader application than bone metastatic prostate cancer. Identification of the proteins which are associated with cell invasion and aggressive disease and whose degradation is controlled by FBXL4, such as ERLEC1, has the potential to develop targeted therapies for a large number of lethal human cancers with loss of *FBXL4*.

## Materials and Methods

### Patient cohort

Six fresh frozen and 43 FFPE prostate cancer bone metastases were collected between 2007–2012 from The Robert Jones and Agnes Hunt Orthopaedic Hospital in Oswestry, Shropshire. All prostate cancer bone metastases came from patients with advanced prostate cancer who were treated for pathological bone fractures. Four fresh frozen primary prostate cancer and four BPH samples as well as FFPE prostate samples, including 145 primary tumours and 55 BPH cases, were obtained from Bartshealth NHS Trust in London. Peripheral blood samples for CTC isolation were collected from ten prostate cancer patients from Bartshealth NHS Trust, London, UK. Tissue microarray sections of FFPE cases of conservatively managed localised prostate adenocarcinomas diagnosed by TURP and selected through six UK Cancer Registries (TAPG cohort) were used for prognosis correlation. National approval for TAPG cohort was obtained from the Northern Multi-Research Ethics Committee, followed by local ethics committee approval at each of the collaborating hospital trusts^2^. The other tissue and blood samples were collected and used for this study under ethical approval from the East London & the City Research Ethics Committee. Informed written consent was obtained for all tissue samples collected prospectively, including all blood samples for CTC analysis. Anonymised retrospectively collected samples were obtained from Orchid Tissue Bank (HTA license number: 12199). All tissue samples were reviewed by consultant pathologist (Dan Berney) and marked for areas of cancer and BPH. All experiments were carried out in accordance with the approved guidelines.

### Cell lines

Five human prostate cancer cell lines, PC-3 (from a bone metastasis), DU-145 (from a brain metastasis), LNCaP (from a lymph node metastasis), VCaP (from a vertebral bone metastasis), and 22RV1 (prostate carcinoma), one human osteoblast-like MG63 cell line (osteosarcoma-derived) (ATCC, Manassas, VA, USA) and commercial The Tetracycline-Regulated Expression (T-Rex) HEK293 cell line (embryonic kidney cells, which stably express the tetracycline (Tet) repressor and are designed for use with the T-REx™ System, Life technology, UK) were used in this study and maintained in Dulbecco’s MEM (DMEM) (Sigma Aldrich, UK) containing 10% fetal calf serum and 1% penicillin/streptomycin (100 units/ml, Sigma Aldrich, UK). All cell lines were verified by microsatellite short tandem repeat (STR) profiling using the ABI AmpF/STR Identifiler kit (Life technology, USA).

### SNP array analysis

A standard phenol/chloroform method was used to extract DNA from fresh frozen bone metastases. Six fresh-frozen prostate cancer bone metastases samples were prepared for hybridisation onto Affymetrix SNP 6.0 array chips (Affymetrix, Santa Clara, CA, USA). The samples were processed according to the manufacturer’s protocols. All standard quality control steps recommended by manufacturer were also followed in the process. Signal intensity data from SNP arrays were analysed using our in-house GOLF (V2.2.10) software^47^.

### FISH analysis

FISH on tissue sections were performed as previously described^47^. Briefly, FFPE tissue slides were de-waxed, rehydrated and boiled in tissue pre-treatment solution for 15 min, followed by 5 min enzyme digestion (SPOT-Light Tissue Pre-treatment kit, Invitrogen, Life Technologies, UK). Total of 1 μl of labelled probes for each chromosomal locus was mixed with 10 μl of hybridisation buffer and applied on pre-treated slides. Slides were then coverslipped, denatured at 95 °C for 10 min, and incubated overnight at 37 °C. Slides then underwent standard post-hybridisation washes at 42 °C with 2 × 50% formamide/2x SSC and 2x SSC, 5 min each, and were incubated with the streptavidin-Cy3 conjugate (Sigma, UK) followed by anti-DIG-FITC antibody (Roche, UK) at 37 °C for 10 min. Eventually, slides were coverslipped with Vectashield antifade containing DAPI (Invitrogen, Life Technologies, UK).

For CTCs, cells after immunofluorescence staining were fixed by methanol: acetic acid 3:1 and washed with 70% acetic acid for 10 min to remove immunofluorescence signals. Bacterial artificial chromosomes (BACs) RP11–639P13, CTD-2073H5, RP11–258I9 and CTD-2281M23 were used in combination for *FBXL4* region and paracentromere of chromosome 1 and 6p12 region (RP11–727H16) were used as controls. The BAC clones were obtained from the Institute of Cancer Research (Sutton, UK). BAC DNA labelling was done with BioPrime® DNA Labelling System (Invitrogen, Life Technologies, UK) according to the manufacturer protocol. Probes were labelled using either biotin-14-dCTP or digoxigenin (DIG)-11-dUTP (Roche, UK).

Both FISH probe signals were counted per nucleus. A minimum of 100 nuclei (cells) with clear hybridisation signals were counted per tissue sample and for circulating samples, all countable CTCs were included. The percentage of nuclei with less *FBXL4* signals than control signals were determined and the widely accepted cutoff calculated as the mean of false-positive findings in ten non-malignant controls (BPH samples or lymphocytes respectively) plus three times the standard deviation (mean % ± 3 SD) was used for *FBXL4* copy number status evaluation in each sample^48, 49^. FISH scoring was done blindly and results given to the statistician who then analysed them in connection to clinical data.

### Quantitative PCR

Total DNA was extracted from cells and tissue using standard phenol-chloroform protocol. Total RNA was extracted with TRIzol reagent (Invitrogen, Life Technologies, UK) according to manufacturer’s instructions. cDNA synthesis was performed with 1 µg of total RNA per sample using Moloney Murine Leukemia Virus Reverse Transcriptase, RNase H Minus, Point Mutant kit (Promega, UK). All TaqMan assays were performed according to the manufacturer protocols (Life technology, UK). DNA copy number analysis of *FBXL4* genomic locus was performed with TaqMan DNA copy number assay (Life technology, UK). Copy number was analysed with CopyCaller Software v2.0 (Life technology, UK) and normalised to normal male DNA. RNase P TaqMan Copy Number Reference Assay was used as endogenous control (Life technology, UK). Standard q-RT-PCR was performed using predesigned TaqMan gene expression assays targeting *FBXL4* and *BRN2* (Life technology, UK). The *GAPDH* gene was used as endogenous control (Life technology, UK).

### Gene knockdown by siRNA

Cells plated at a density of 7 × 10^4^ (PC3 and DU145) or 12 × 10^4^ (22RV1) per well in six-well plates were transfected with 100 nM of *FBXL4*-specific smart pool siRNA and control non targeting siRNA (GE Dharmacon, Layfayette, CO, USA) using oligofectamine reagent (Life technology, UK) according to the manufacturer’s protocols.

### *FBXL4* overexpression in HEK293 cells

T-REx HEK293 cells were purchased together with a tetracycline-regulated mammalian expression system (the T-REx™ system) from Invitrogen, UK. Full-length *FBXL4* cDNA carrying a 5′-FLAG tag was subcloned into the pcDNA4/TO expression vector and stable pools of HEK293 transfectants were generated by selection with 400 µg/ml of zeocin. HEK293 cells transfected with empty pcDNA4/TO vector were used as a control. Induction of *FBXL4* expression from pcDNA4/TO expression vector was done with 1 µg/ml of tetracycline treatment for 48–72 h.

### Western Blotting

Western blotting was performed as previously described^50^ using anti-FBXL4 (rabbit polyclonal - ab153812, Abcam, UK), anti-ERLEC1 (rabbit polyclonal - ab102046, Abcam, UK), anti-FAK (mouse monoclonal - 05–537, Millipore, UK), anti-phospho-FAK (Y397, rabbit monoclonal - ab81298, Abcam, UK), anti-N-cadherin (mouse monoclonal - ab98952, Abcam, UK), anti-vimentin (rabbit monoclonal - AC-0024, Epitomics, UK) and anti-CK18 (mouse monoclonal - NCL-CK18, Novocastra, UK) antibodies. 30–50 μg of whole cell protein lysates in RIPA buffer were mixed with NuPAGE® LDS Sample Buffer (BioRad, UK) and reducing agent (BioRad, UK) and denatured at 90 °C for 10 min.

Protein samples were separated on 10% polyacrylamide gels and transferred onto PVDF membranes (Millipore, UK). Membranes were next blocked with 5% powder milk in Tris Buffer Saline/0.1% Tween-20 (TBST) and incubated overnight with primary antibodies, followed by secondary peroxidase conjugated antibody (Fisher Scientific, UK) for 1 h at room temperature. Protein detection was done with Immobilon Western Chemiluminescent HRP Substrate (Millipore, UK). β actin levels were used as a loading control (mouse monoclonal - A5441, Sigma-Aldrich, UK).

### CTC isolation and immunofluorescence staining

CTCs were isolated and immunofluorescence staining was performed on CTC slides as previously described^20, 51^. Briefly, CTCs from 7.5 ml of peripheral blood samples from prostate cancer patients were isolated within 1–24 h after blood draw with Parsortix system (ANGLE PLC, UK). Parsortix system uses a micro-fluidic cassette to capture and harvest CTCs based on their less deformable nature and larger size compared to other blood components. Captured cells were eluted from the cassette with 200 µl of buffer and transferred onto a glass slide. Cell solution was then air-dried, followed by 20 min cell fixation with 100% acetone on ice.

Immunofluorescence staining was performed using rabbit polyclonal anti-CD45 (Santa Cruz, USA) and mouse monocolonal anti-cytokeratin (CK) (Miltenyi Biotec, UK) antibodies. Eventually, CTC slides were mounted in SlowFade® gold antifade mountant with DAPI. Stained slides were scanned using Ariol image analysis system (Leica Microsystems Ltd, Suffolk, UK). A CTC is defined as a nucleated cell with CK+ and CD45−.

### Cell viability, migration and invasion assays

Cell viability and proliferation were assessed using the CellTiter 96 AQueous Cell Proliferation Assay (MTS) (Promega, UK) following the manufacturer’s instructions. 3000–5000 cells (depending on cell line) were plated in each well of a 96-well plate. On the following day, prostate cancer cell lines were transfected with anti-FBXL4 siRNA and after 48 h and 72 h, cell viability and proliferation were assessed by MTS assay. T-Rex HEK293 cells were treated with 1 µg/ml tetracycline for 42 h prior to MTS assay to ensure *FBXL4* overexpression. *In vitro* transwell migration and Matrigel invasion assays were performed by using 24-well, 8-µm pore transwell inserts (Becton Dickinson, UK). 48 h post transfection with siRNA, 3 × 10^4^–7 × 10^4^ prostate cancer cells were seeded in 200 µl of serum-free growth medium in the upper chamber, and 600 µl of medium with chemo-attractant (10% FCS) was added to the lower chamber. For PC3 cells, MG63 human osteoblasts cells were used as chemo-attractant. MG63 were seeded at the bottom of 24 well plate wells and 24 h before experiments old growth medium was removed and replaced with 600 µl of plain DMEM. T-Rex HEK293 cells were treated with 1 µg/ml tetracycline for 48 h prior to transwell migration assay to ensure *FBXL4* overexpression. In all experiments prostate cancer cells were incubated at 37 °C for 24 h, and then fixed in 10% buffered formalin and stained with haematoxylin (Fisher Scientific, UK). Cells on the upper surface were removed with a cotton swabs, and migrated cells on the underside were counted (average of 10 fields). For collagen migration/invasion assay cells 48 h post transfection with *FBXL4* and control siRNA were seeded onto collagen (2 mg/ml) as a single cell suspension. 24 h later, cells with pseudopodia-like extensions were counted as migrating/invading cells^52^. Number of migrating/invading cells was determined by counting the cells in at least 10 random fields.

### Statistical analysis

Statistical tests included the chi-squared (*X* ^2^) for FISH data comparison and correlation of *FBXL4* status with Gleason score and prostate cancer clinical stage, Mann-Whitney U tests for *FBXL4* status correlation with PSA or extent of disease, Kruskal-Wallis test for q-RT-PCR data and paired Student’s *t* -test, for functional studies. Statistical analyses were performed using the Prism 5.0b (GraphPad, La Jolla, CA, USA) software. Values of p < 0.05 were considered significant.

Statistical analysis of TAPG cohort was done with STATA (version 11.2, StataCorp, USA). The primary end point was time to death from prostate cancer, assessed with a proportional hazards model. Observations were censored on the date of last follow-up, or at death from other causes. For the multivariate Cox proportional hazards models, forward stepwise regression was used. Covariates evaluated were Gleason score, baseline PSA value, clinical stage, extent of disease (proportion of positive TURP chips), age at diagnosis and *FBXL4* status (*FBXL4* loss versus no loss). All p-values were two-sided and p values of 0.05 or less were considered significant.

## Electronic supplementary material

Supplementary Information
