# Supplementary material for: Identification of FBXL4 as a Metastasis Associated Gene in Prostate Cancer
Source: Sci Rep. 2017 Jul 11;7:5124. doi: 10.1038/s41598-017-05209-z (PMC5505985; doi:10.1038/s41598-017-05209-z)
Supplement: Supplementary file 1 — Supplementary Information [file 41598_2017_5209_MOESM1_ESM.pdf]

Supplementary Files

Identification of *FBXL4* as a Metastasis Associated Gene in Prostate Cancer

Elzbieta Stankiewicz, Xueying Mao, D Chas Mangham, Lei Xu, Marc Yeste-Velasco, Gabrielle Fisher, Bernard North, Tracy Chaplin, Bryan Young, Yuqin Wang, Jasmin Kaur Bansal, Sakunthala Kudahetti, Lucy Spencer, Christopher S Foster, Henrik Moller, Peter Scardino, R Tim Oliver, Jonathan Shamash, Jack Cuzick, Colin S Cooper, Daniel M Berney, Yong-Jie Lu

Supplementary Figures

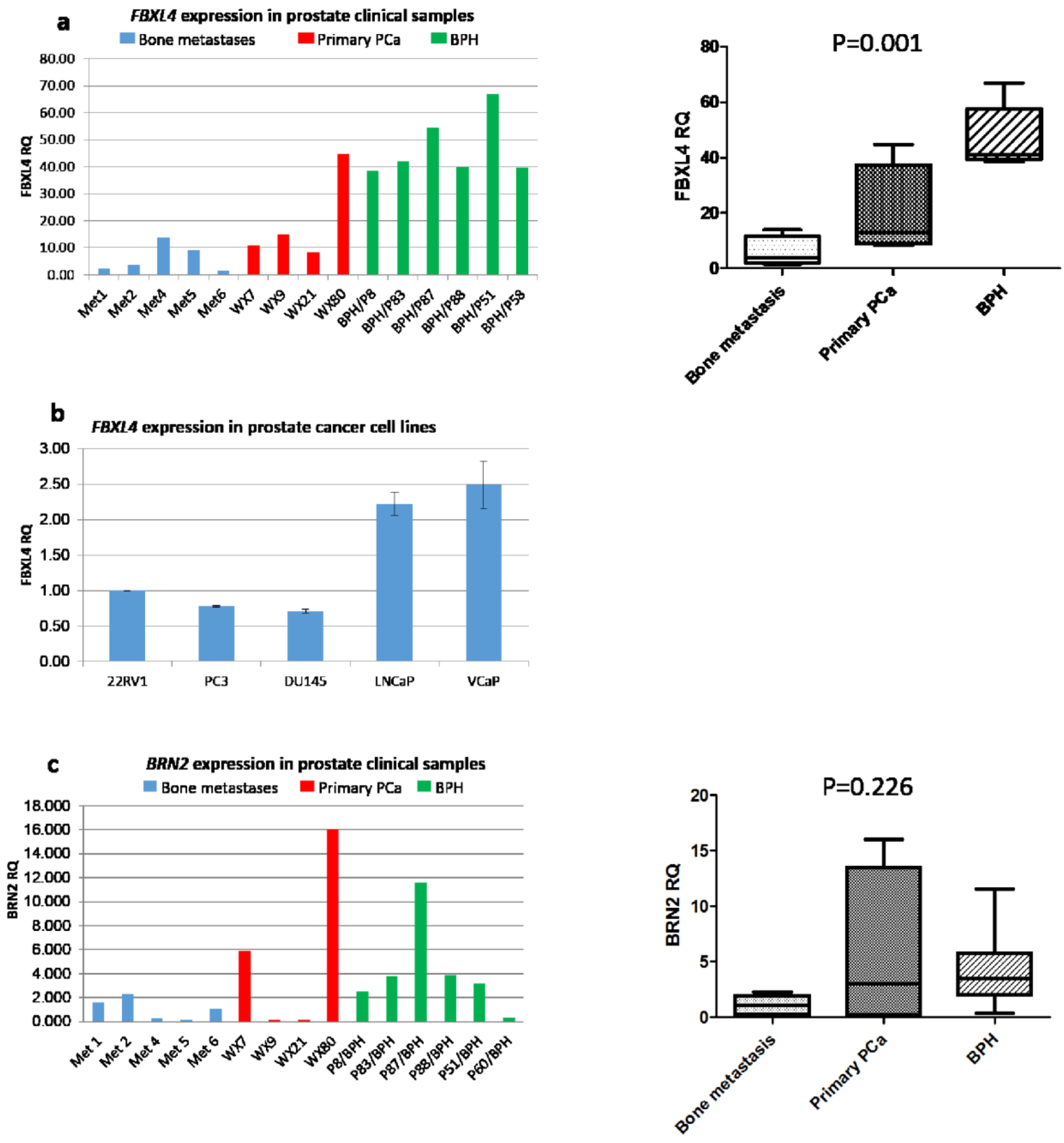

Supplementary Figure 1. RNA expression of *FBXL4* and *BRN2* in prostate clinical samples and cell lines. (**a**) Clear reduction in *FBXL4* RNA expression levels was detected in prostate cancer bone metastases (Met1-6) and primary tumours (WX cases) when compared to BPH samples (p=0.001, Kruskal-Wallis test), implying *FBXL4* gene as a driver for 6q16 deletion. (**b**) *FBXL4* expression in prostate cancer cell lines. PC3 and DU145 cells harbouring heterozygous 6q16 loss show reduced

FBXL4 RNA levels when compared to cells without 6q16 loss. (**c**) There is no difference in *BRN2* expression between prostate cancer samples and non-malignant BPH cases suggesting that this gene is not a driver for the 6q16 loss (p=0.226, Kruskal-Wallis test). Met1-6, prostate cancer bone metastases; WX7-80, primary prostate cancer; BPH, benign prostatic hyperplasia; PCa, prostate cancer.

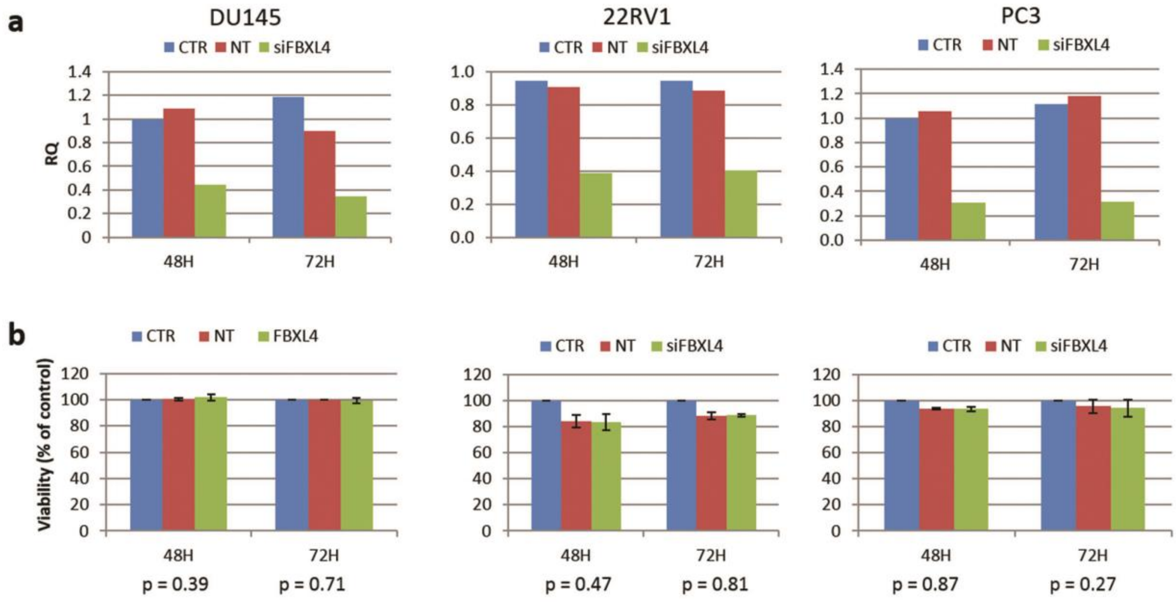

Supplementary Figure 2. The effect of *FBXL4* knockdown on prostate cancer cells viability. (**a**) Confirmation of *FBXL4* knockdown at mRNA level in DU145, 22RV1 and PC3 cells. (**b**) *FBXL4* knockdown does not affect prostate cancer cell viability in DU145, 22RV1 and PC3 cells compared to non-targeting siRNA. Cell viability was measured by MTS assay at 48 and 72h post *FBXL4* siRNA transfection. Ctr, non-transfected control cells; NT, cells transfected with non-targeting siRNA; siFBXL4, cells transfected with *FBXL4* siRNA. Error bars indicate SD; n = 3.

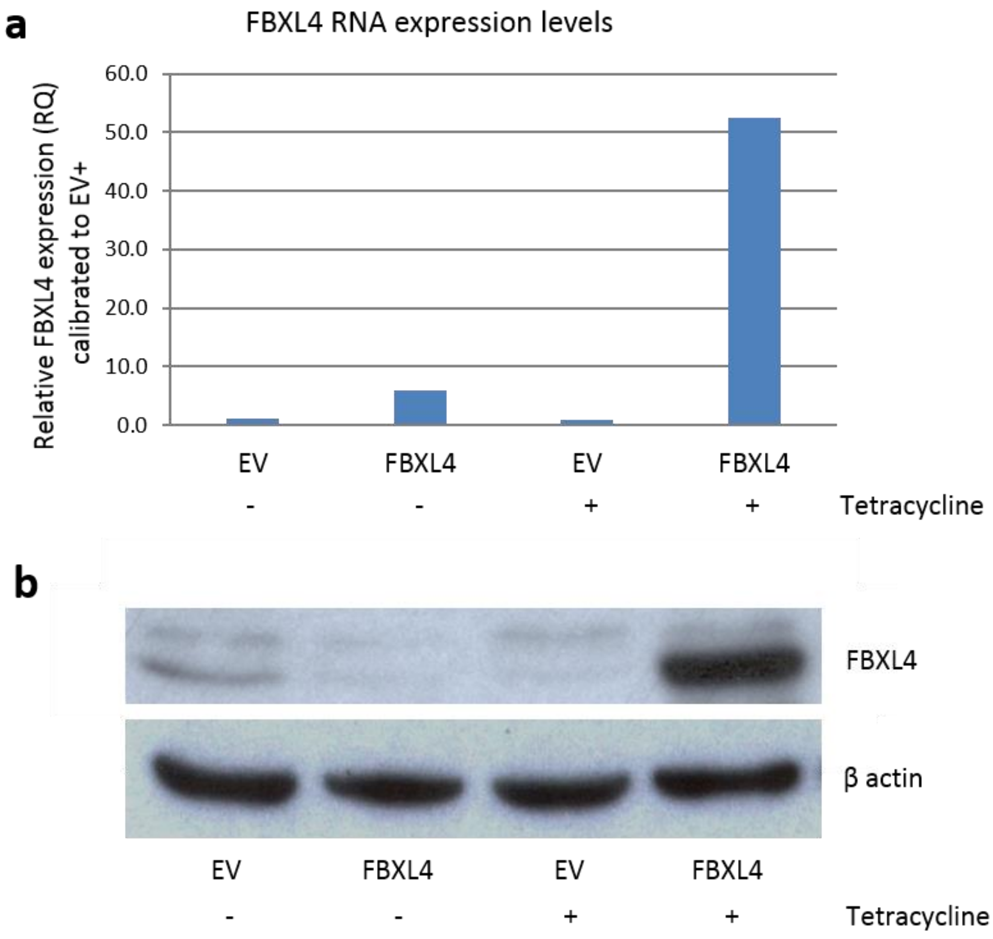

Supplementary Figure 3. Confirmation of induced *FBXL4* overexpression in HEK293 cells. (**a**) *FBXL4* RNA expression after 24 h treatment with/without 1µg/ml tetracycline. **(b**) FBXL4 protein levels after 72 h treatment with/without 1µg tetracycline. *FBXL4* overexpression is clearly present in tetracycline treated HEK293 cells containing *FBXL4* expression vector when compared to control HEK293 cells or untreated cells. EV, control cells with empty expression vector; FBXL4, HEK293 cells transfected with *FBXL4* expression vector. ‘-‘, no tetracycline treatment; ‘+’, 1µg/ml tetracycline treatment.

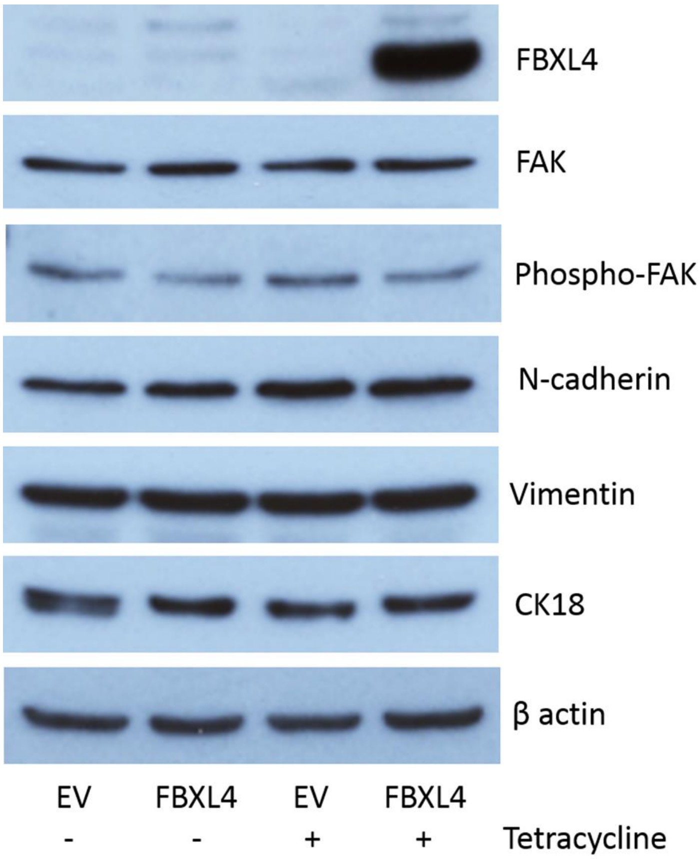

Supplementary Figure 4. *FBXL4* overexpression does not affect certain EMT and cell migration promoting markers in HEK293 cells. No reduction in the levels of crucial for cell attachment and migration total and activated (phosphorylated) focal adhesion kinase (FAK), as well as mesenchymal markers such as N-cadherin and vimentin, and no increase in epithelial cytokeratin 18 (CK18) were observed by western blot in HEK293 cells with tetracycline-induced overexpression of FBXL4 protein (FBXL4+) when compared to control cells. Protein load: 50 µg. EV, control cells with empty expression vector; FBXL4, HEK293 cells transfected with expression vector containing *FBXL4*. '-', no tetracycline treatment; '+', 1 µg/ml tetracycline treatment for 72 h.

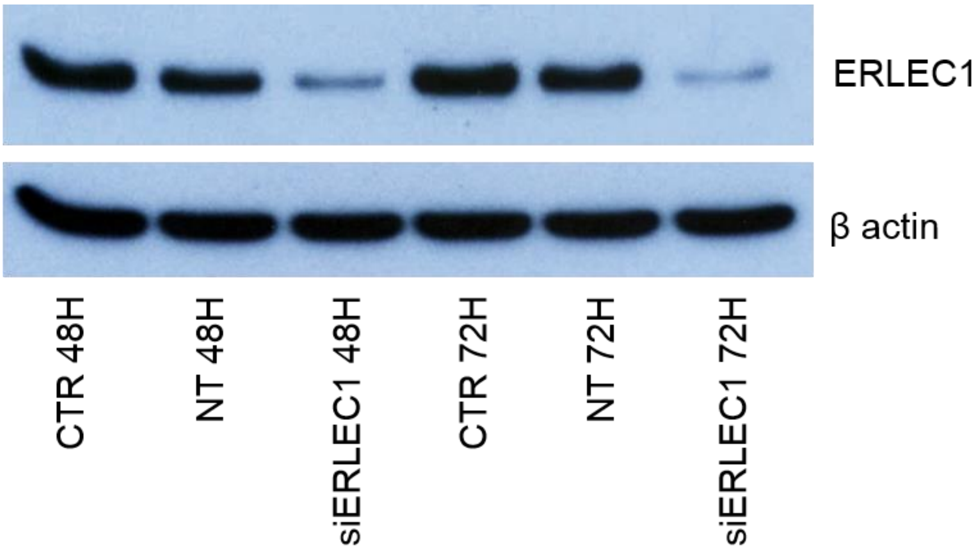

Supplementary Figure 5. Confirmation of anti-ERLEC1 antibody specificity using in vitro knock down model. HEK293 cells were transfected with anti-ERLEC1 siRNA (siERLEC1) and control non-targeting siRNA (NT). The whole cell lysates were collected after 48 h and 72 h for western blot analysis. Mock-transfected cells (CTR) were used as additional control. A specific band at the predicted ERLEC1 protein size was detected and the intensity of the band was dramatically reduced in cells with ERLEC1 knockdown, confirming the specificity of the antibody (ab102046, Abcam, UK).

Supplementary Tables

Supplementary Table 1. Frequency of *FBXL4* loss detected by FISH in primary and metastatic prostate cancer.

**Prostate cancer types**	***FBXL4* copy number loss**		**p value (X^2 test)**
	**positive**	**negative**	
Primary	20/145 (13.8%)	125/145 (86.2%)	0.0003
Bone metastases	11/23 (47.8%)	12/23 (52.2%)	

Supplementary Table 2. Multivariate survival correlation analysis of *FBXL4* loss and clinicopathological parameters in localised prostate cancer.

**Parameter**	**HR (95% CI)**	**P value**
FBXL4 loss	0.75	0.25
Gleason score <7 vs. =7	0.75	0.25
Gleason score >7	5.58	0.000002
PSA	1.37	0.0075
Clinical stage T1	0.70	0.34
Clinical stage T2	0.96	0.90
Clinical stage T3	0.96	0.89
Extent of the disease	3.88	0.0001

Supplementary Table 3. CTC *FBXL4* FISH results and associated prostate cancer patient clinical data.

Sample ID	PSA (ng/ml)	Gleason Grade	CRPC* Y/N	mCRPC** Y/N	All CTC counting	*FBXL4* loss
PC19b	40	No data	Y	Y	11	+
PC40	21	4+4	N	N	33	+
PC42	No data	No data	Y	N	21	+
PC49	17	4+3	Y	Y	14	-
PC56	24	5+5	Y	Y	12	+
PC59	17	4+4	Y	Y	25	+
PC60	5230	4+4	Y	Y	16	+
PC61	1500	n/a	Y	Y	10	+
PC69	202	5+5	Y	Y	10	+
PC81	No data	3+3	N	N	4	-

*castration resistant prostate cancer

**metastatic castration resistant prostate cancer

Supplementary Table 4. Increased migration and invasion in prostate cancer cell lines with *FBXL4* knockdown by siRNA.

Prostate cancer cell line		Transwell migration	Matrigel invasion	Collagen invasion
		Increase in relation to NT cells (%)		
**DU145**	**mean**	127.6	155.2	15.3
	**SD**	10.4	14.3	3.0
	**p value** (t-test*)	0.04	0.001	0.01
**22RV1**	**mean**	146.5	175.8	13.5
	**SD**	10.2	30.4	2.4
	**p value** (t-test*)	0.02	0.0498	0.01
**PC3**	**mean**	124.7	133.3	9.8
	**SD**	1.7	5.1	2.9
	**p value** (t-test*)	0.002	0.0001	0.03

*all values were compared to NT cells (controls transfected with non-targeting siRNA)
